# Septic shock due to *Pasteurella multocida* bacteremia: a case report

**DOI:** 10.1186/s13256-015-0643-3

**Published:** 2015-07-11

**Authors:** Niyati Narsana, Faria Farhat

**Affiliations:** Department of Medicine, Medstar Washington Hospital Center, 110 Irving street NW, Suite 2A-50, Washington, DC 20010 USA

**Keywords:** Bacteremia, Cat scratch, *Pasteurella multocida*, Septic shock

## Abstract

**Introduction:**

*Pasteurella* is a Gram-negative coccobacillus that causes a wide spectrum of diseases in humans and is commonly transmitted from cat and dog bites. An increasing number of cats and dogs are kept as pets in American households which increases the risk of pet-related infections.

**Case presentation:**

An 82-year-old African American woman with multiple comorbidities presented with fever, vomiting and diarrhea; she later developed septic shock requiring vasopressors and intubation. She was found to have *Pasteurella multocida* bacteremia. Her hospital course was complicated by a pulseless electrical activity arrest. She had exposure to her pet cat at home. We believe that a possible portal of entry was her skin; however, other possibilities such as respiratory tract could not be excluded. She was treated with imipenem-cilastatin and discharged after 25 days.

**Conclusions:**

Studies have shown a mortality range from 7 to 31% in *Pasteurella* bacteremia. Due to an increasing number of pets and high mortality of this disease, it is important to have a high suspicion for this infection, especially in elderly and immunocompromised patients.

## Introduction

*Pasteurella multocida* is small Gram-negative coccobacillus that is a component of the upper respiratory tract and gastrointestinal flora of many animals [1]. Human infections are most commonly caused by cat and dog bites. *Pasteurella* is the most common organism isolated from cat and dog bites [2].

According to the recent ‘National Pet Owners Survey’ of the American Pet Products Manufacturers Association, 42 million and 54 million US households own cats and dogs as pets respectively [3]. There are approximately 85 million cats and 77 million dogs owned as pets in America [3]. Every year, there are approximately 300,000 emergency department visits in America due to animal bites [2].

*Pasteurella* can cause a wide spectrum of diseases from local infections to septic shock. We present a case of septic shock in an elderly woman due to *Pasteurella multocida*.

## Case presentation

An 82-year-old African American woman presented to our emergency department with a 2-hour history of non-bilious, non-bloody vomiting, and one episode of loose stools. She later developed worsening shortness of breath and was found to be febrile to 39.7°C, pulse 104 beats/minute, blood pressure 169/71mmHg, respiratory rate of 18 breaths/minute and saturating 95% on room air. She was obese with body mass index 35 and her examination was significant only for some bibasilar crackles. She denied any travel history, contact with people who were ill, abdominal pain, chest pain or dysuria.

**Fig. 1:**
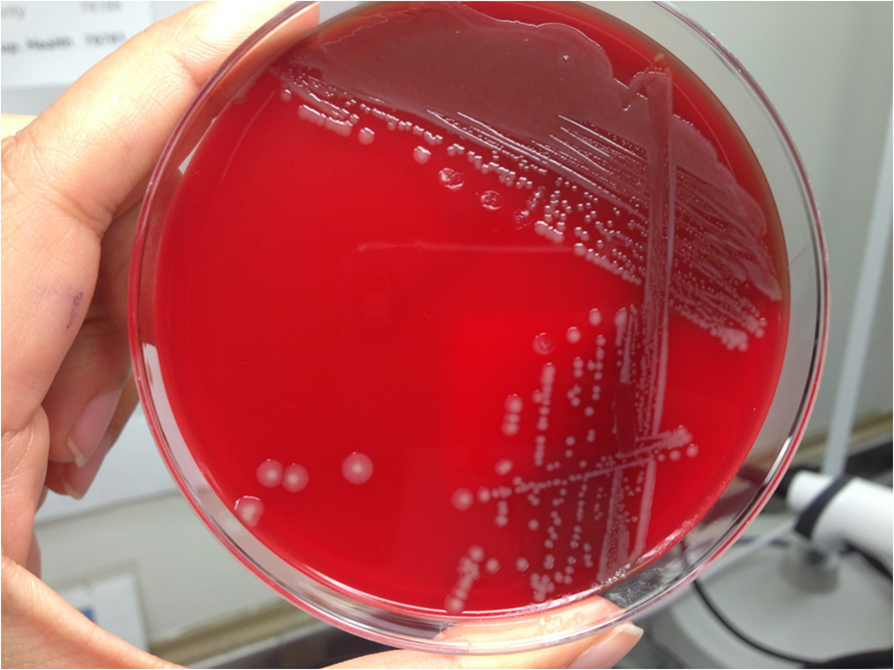
Grey non-hemolytic colonies of *Pasteurella multocida* on blood agar. *Pasturella multocida* is aerobic and facultative anaerobic and grows on blood agar, chocolate agar but not on MacConkey’s agar. Most isolates are oxidase, indole and catalase positive

**Fig. 2:**
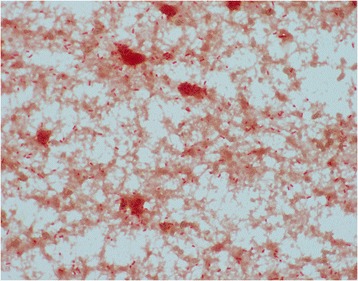
Non-motile Gram-negative rods of *Pasteurella*. Gram-negative rods of *Pasteurella* as seen under the microscope

Her past medical history was significant for hypertension, hyperlipidemia, chronic obstructive pulmonary disease (COPD), coronary artery disease status post-coronary artery bypass, stroke, and breast cancer status post-chemotherapy and radiation therapy. Laboratory test results on admission were remarkable for white blood cell count (WBC) of 15,500, lactic acid of 2.5mmol/l, creatinine of 1.19mg/dl which was her baseline, and negative troponins. An electrocardiogram showed normal sinus rhythm. Computed tomography (CT) of her abdomen-pelvis was unremarkable. Her chest X-ray showed cardiomegaly with some pulmonary venous congestion. The suspicion for sepsis was high; however, the source was unclear at that time. Blood and urine cultures were sent, and on an empirical basis vancomycin and piperacillin-tazobactam were administered intravenously. Her lactic acid increased to 5mmol/l, creatinine to 2.28mg/dl and WBC to 27,000 in a few hours. She became hypotensive requiring pressor support and was admitted to our intensive care unit for possible septic shock. Overnight, she had a cardiopulmonary arrest with pulseless electrical activity and there was a return of spontaneous circulation after chest compressions and epinephrine in 5 minutes. She was intubated for hypoxic respiratory failure. Her arterial blood gas showed pH of 7.14, partial pressure of oxygen (pO_2_) of 98mmHg, partial pressure of carbon dioxide (pCO_2_) of 43mmHg and bicarbonate of 15mmol/l. She received alteplase and was started on heparin drip for possible pulmonary embolism. On the second day, two blood cultures grew non-motile Gram-negative rods and vancomycin was stopped. Due to the worsening respiratory status and suspicion for ventilator-associated pneumonia and extended-spectrum beta-lactamases, piperacillin-tazobactam was switched to imipenem-cilastatin. The colonies of the organism grew on blood and chocolate agar but not on MacConkey agar. The growth on blood agar was gray and non-hemolytic (Figs. 1 and 2).

It was catalase, oxidase and indole positive and a RapID NH test (a qualitative test to identify species of *Neisseria*, *Haemophilus* and other related microorganisms isolated from humans) confirmed it as *Pasteurella multocida*. Her bronchial brush, sputum and urine cultures remained negative.

On careful examination, there were scratch marks and abrasions on her left leg. On further history, her daughter mentioned that her cat died a few days ago, and she bought another cat after that. We believe that the most likely route of transmission of *Pasteurella* infection was through the skin; however, another route could not be ruled out. She was eventually weaned off the pressors and was extubated after 7 days. The repeat blood cultures were negative. Her hospital course was complicated by hematemesis leading to aspiration, repeat cardiopulmonary arrest and re-intubation. At that time, she received steroids for possible COPD exacerbation. She also developed *Clostridium difficile* colitis, and was treated with oral vancomycin for 14 days. She was extubated successfully after 3 days, and was treated with imipenem-cilastatin for a total of 19 days for *Pasteurella* bacteremia and possible aspiration pneumonia. She was discharged after 25 days of hospitalization to a subacute rehabilitation center.

## Discussion

*Pasteurella* infections in humans commonly result from contact with animals such as cats, dogs, swine, lions, panthers, horses, rats, rabbits, and wolves [4–6]. It is a common commensal organism found in the oropharynx. Cats and dogs have a colonization rate of approximately 70 to 90% and 20 to 50% respectively [4, 6, 7]. Animal bites and scratches are the most common modes of acquisition of infection in humans. Cases of infection without any animal exposure have also been reported [4, 8].

*Pasteurella* is a non-motile aerobe and facultative anaerobe, which grows on chocolate and blood agar, but not on MacConkey agar. *Pasteurella multocida* does not cause hemolysis on blood agar, and grows in carbon dioxide-rich medium at 37°C [4, 5].

It can involve skin and soft tissue, bone and joint, upper and lower respiratory tract, and cause more severe infections such as meningitis, bacteremia, endocarditis and peritonitis. Local cutaneous infections are the most common [5, 9, 10]. In a study by Escande and Lion, bacteremia was found in 11% of 958 cases of *Pasteurella* infections [10]. In a study by Ebright *et al.* at Detroit Medical Center, 7.8% of 179 patients had positive blood cultures [9]. The various sources of isolation of this organism reported in the literature are local wounds, sputum, bronchoalveolar lavage, cerebrospinal, pleural, ascitic and joint fluid [5, 9, 10]. There are many cases where the source of bacteremia is unknown [10, 11]. Our patient had the organism isolated only from her blood and the possible route of entry was a cat scratch. However, other possible routes of entry such as the respiratory tract could not be confirmed as bronchial brush cultures were negative.

*Pasteurella* infections are more common in elderly patients with underlying chronic diseases such as diabetes mellitus, hypertension, cardiac disease, acquired immunodeficiency syndrome (AIDS), malignancies, COPD, cirrhosis and dialysis [9, 10, 12]. The study by Escande and Lion showed 70% of systemic infections in ages >50 years and 21% in ages 20 to 50 years [10]. Our patient had multiple comorbidities including hypertension, hyperlipidemia, COPD and coronary artery disease which made her more susceptible to developing this severe infection.

Most of the studies have shown susceptibility of *Pasteurella multocida* to penicillins, beta-lactams, carbapenems, second and third generation cephalosporins and tetracyclines [5, 12–14]. Severe systemic infections are treated with intravenous antibiotics and local infections with oral agents [9, 10]. Studies have shown a mortality ranging from 7 to 31% in patients with *Pasteurella* bacteremia [9–11].

## Conclusions

There is an increasing number of pets in American households leading to increased exposure to animals. This can increase the risk of developing infections, especially in the elderly and immunocompromised population. It is important to maintain appropriate hygiene to prevent transmission of infection from pets. Given the high mortality of this disease and increasing number of pets, it is important to have a high suspicion for pet-related infections such as *Pasteurella multocida*.

## Consent

Written informed consent was obtained from the patient for publication of this case report and accompanying images. A copy of the written consent is available for review by the Editor-in-Chief of this journal.

## Abbreviations

COPD: Chronic obstructive pulmonary disease
WBC: White blood cell count
